# Automatic Dissolution Testing with High-Temporal Resolution for Both Immediate-Release and Fixed-Combination Drug Tablets

**DOI:** 10.1038/s41598-019-53750-w

**Published:** 2019-11-19

**Authors:** Zhongmei Chi, Irfan Azhar, Habib Khan, Li Yang, Yunxiang Feng

**Affiliations:** 1 Faculty of Chemistry, Northeast Normal University, 5268 Renmin Street, Changchun, Jilin 130024 P.R. China; 2 Jingke-Oude Science and Education Instruments Co., Ltd., Changchun, Jilin 130024 P.R. China

**Keywords:** Analytical chemistry, Drug development

## Abstract

Dissolution testing plays many important roles throughout the pharmaceutical industry, from the research and development of drug products to the control and evaluation of drug quality. However, it is a challenging task to perform both high-efficient separation and high-temporal detection to achieve accurate dissolution profile of each active ingredient dissolved from a drug tablet. In our study, we report a novel non-manual-operation method for performing the automatic dissolution testing of drug tablets, by combining a program-controlled sequential analysis and high-speed capillary electrophoresis for efficient separation of active ingredients. The feasibility of the method for dissolution testing of real drug tablets as well as the performance of the proposed system has been demonstrated. The accuracy of drug dissolution testing is ensured by the excellent repeatability of the sequential analysis, as well as the similarity of the evaluation of dissolution testing. Our study show that the proposed method is capable to achieve simultaneous dissolution testing of multiple ingredients, and the matrix interferences can be avoided. Therefore it is of potential valuable applications in various fields of pharmaceutical research and drug regulation.

## Introduction

Dissolution testing determines the rate and extent at which a drug is released from its pharmaceutical dosage form^1^. It plays many important roles throughout the pharmaceutical industry, including drug product research and development, controlling and evaluating drug quality, and assessing the quality and efficacy consistency of generic drugs. *In vitro* dissolution testing is a primary test in the pharmaceutical industry^2–5^. In order to establish *in vitro* and *in vivo* correlations (IVIVCs) of drugs, as well as to evaluate dosages for compliant with quality standards, accurate information about dissolution kinetics (i.e., time-dependent dissolution) is strongly required^6–11^.

A methodology for dissolution testing should be capable to trace the evolution of any active pharmaceutical ingredients in a drug from the beginning to the end of the dissolution process^12,13^. This requires both high-temporal resolution and high-efficient separation. The former is important to obtain the dissolution kinetics of immediate-release drug tablets which dissolve so rapidly, while the latter is a pivotal requirement for dissolution testing of fixed-combination drug tablets which have two or more active pharmaceutical ingredients. It is quite difficult for manual off-line approach to achieve high-temporal dissolution testing, since in this approach aliquots are taken from a dissolution apparatus at various time points during dissolution followed by filtration of the dissolved portion and chemical analysis (spectrophotometry, HPLC, etc.)^11,14–19^. Recent advanced automatic on-line methods for dissolution testing based on optical fibers, imaging techniques or voltammetry^20–26^ can obtain real-time dissolution kinetics. These approaches, however, are usually performed without filtration and separation of substances, making them challenging due to the effects of particulates, excipients and scattering, particularly for fixed-combination drugs. Even with mathematical “filters” that include baseline corrections and derivative spectroscopy to remove the contribution of non-active pharmaceutical ingredient components, it is still a challenging task for simultaneous determination of multiple active pharmaceutical ingredients in the same drug tablet, which may have overlapping spectra^27–30^.

Here we propose a novel non-manual-operation method for performing the automatic and high-temporal dissolution testing of drug tablets based on high-speed capillary electrophoresis (HSCE). The method relies on a program-controlled sequential analysis of drug dissolution, integrating a flow-through-cell of pharmacopoeial dissolution apparatus, an automatic sequential sample injection for high-temporal resolution analysis and HSCE for efficient separation and detection. Rapid and sensitive on-line measurements of the dissolution processes of oral tablet drugs can be achieved with a temporal resolution of 20 s. The limitations caused by matrix interferences and simultaneous determination of multiple ingredients in a drug are overcome due to the efficient separation facilitated by HSCE^31–35^. Similarity evaluation of the dissolution profiles of the drug tablets shows the accuracy of the proposed method. Our study is of potential valuable applications in various fields of pharmaceutical research and drug regulation.

## Automatic Dissolution Testing

**Figure 1:**
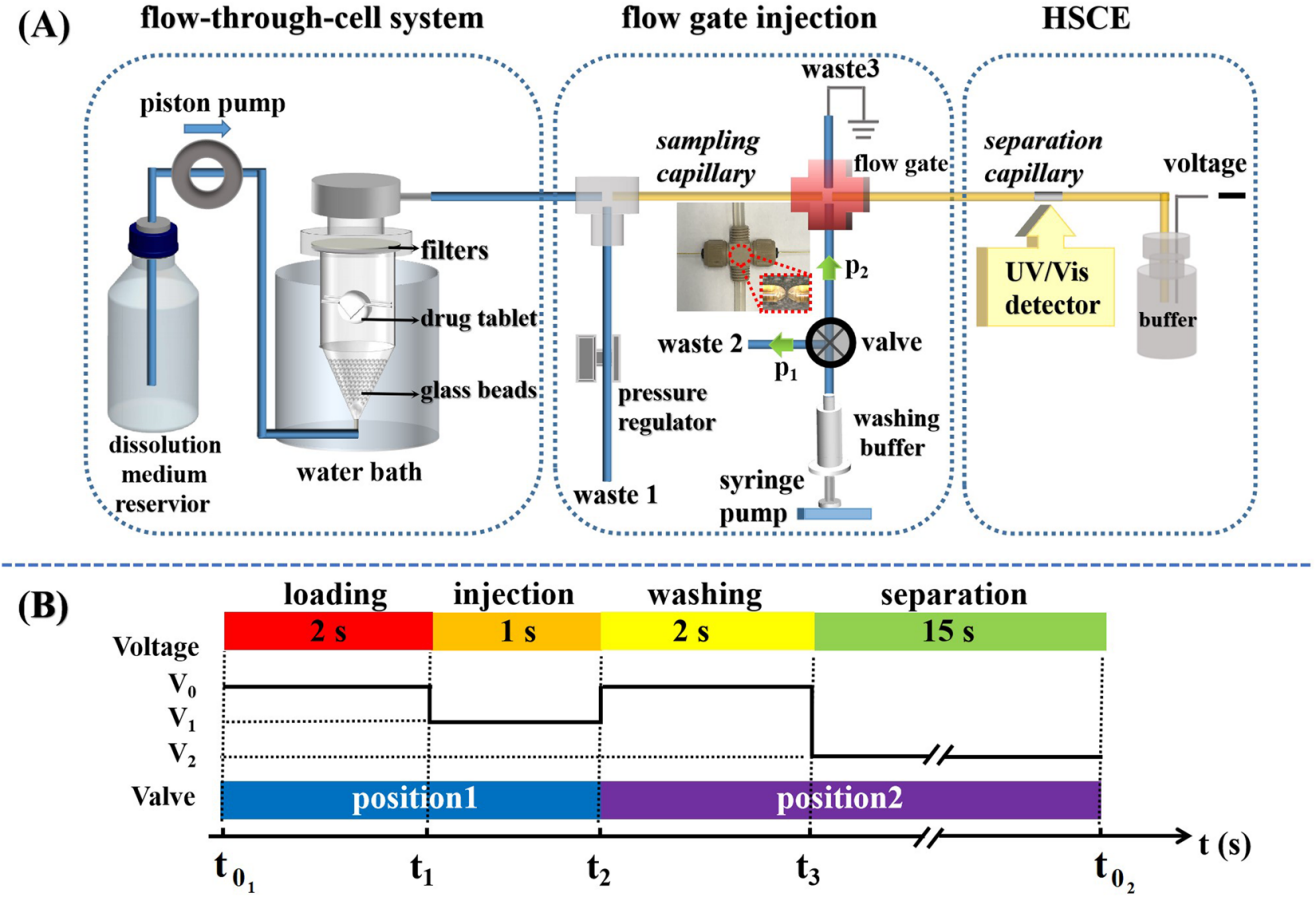
(**A**) Schematic diagram of the proposed system for automatic dissolution testing, which contains of three components: a flow-through-cell of pharmacopoeial dissolution apparatus, an automatic sequential injection with high-temporal resolution and HSCE for on-line separation/detection. (**B**) Diagram of the running sequence for on-line analysis. V_0_ = 0 kV; V_1_ = −5 kV; V_2_ = −15 kV.

Figure 1A shows a diagram of the proposed system for automatic dissolution testing; it composed of three components, i.e., a flow-through-cell for tablet dissolution, an automatic sequential injection with high-temporal resolution and on-line separation/detection using HSCE. The detail information of the system can be found in the Materials and Methods and only a brief description is provided here. A drug was released from tablet in a flow-through-cell containing dissolution medium with the temperature fixed at 37.0 ± 0.5 °C. The dissolution medium carrying the dissolved active ingredients continuously entered the sampling capillary. Sequential injection of the eluted sample was performed via a four-way flow-gating interface^36–39^. The sampling capillary and the separation capillary were tightly inserted into two opposite channels of the interface with a distance of approximately 50 µm (see the inset images in Fig. 1A). The remaining two channels of the interface were fitted with PTFE (polytetrafluoroethylene) tubes for delivering and draining gating buffer solution from the injection space. The flow-gating interface was grounded and a negative high-voltage was applied at the outlet of the separation capillary for HSCE separation and detection.

## Running Sequence of on-line Automatic Dissolution Testing

In our system, the dissolution sample from the flow-through-cell is continuously introduced into the flow-gating interface. Thus, the key issue to achieve on-line dissolution testing with high-temporal resolution using the proposed system is to automatically control the timing of the sequential sample injection and HSCE separation/detection. We develop a LabVIEW program to automatic control the position of the solenoid valve for sample injection and to turn on/off the high voltage power supply for HSCE. Figure 1B shows a diagram of the running sequence for on-line analysis. At t_01_, the solenoid valve (DKST11S6, Shanghai Danke Solenoid Valve Co., Ltd.) is placed at Position 1 (P_1_) to direct the background electrolyte (BGE) flow to waste 2, and the power supply (Tianjin Dongwen High Voltage Power Supply Factory, Tianjin, China) is turned off (V_0_ = 0). In this case, the sample is loaded to the flow-gating interface. As the high voltage V_1_ = −5 kV is applied at time t_1_, the sample is then electrokinetically injected into the separation capillary for HSCE analysis. After a certain injection time, at t_2_, the voltage is turned off again and the valve is set at Position 2 (P_2_) to wash the sample out of the injection space via the BGE. Then, voltage V_2_ (separation voltage) is applied at t_3_ for HSCE analysis. After separation/detection, the valve is turned back to P_1_ and the power supply is turned off, and another sequence of “loading-injection-washing-separation” begins at t_02_. The typical analysis time of one sequence is approximately 20 s, as indicated in Fig. 1B.

## Statistical Analysis

All measurements were performed in triplicate, and the mean and standard deviation were used for statistical analysis. Because the open-loop mode of flow-through-cell system was used in this study, differential dissolution data of drugs were obtained. In order to facilitate the statistical comparison of dissolution profiles, numerical integration method was used to convert differential dissolution data into cumulative dissolution data. The cumulative amount percentage of the drug dissolved (*Dissolved %*) from a tablet in time *t* was used to analyze the dissolution kinetics, which was converted from the experimental measurements using the equation^40^,

1 $$Dissolved\, \% =\mathop{\sum }\limits_{i=1}^{n=\,\max }\frac{({c}_{i-1}+{c}_{i})({t}_{i-1}+{t}_{i})}{2\times D}\times {f}\times 100 \% $$

where *n* is the number of sampling points, *c*_*i*_ is the concentration of drugs in time *t*_*i*_, *D* is the quantity of active components of drugs, and *f* is the dissolution medium flow rate.

Several parameters were calculated to characterize the drug dissolution kinetics. Dissolution Efficiency (*DE%*) was defined as area under the dissolution curve (*AUC*) up to a certain time *t* to that of the area of the rectangle described by 100% dissolution at the same time point (*Q*_100_), and is defined as follows^41^:

2 $$DE\, \% =\frac{AU{C}_{0}^{T}}{{Q}_{100.T}}100$$

The Mean Dissolution Time (*MDT*) was calculated with statistical moments, and are defined by Eq. (3)^42^,

3 $$MDT=\frac{\sum |{{\bf{t}}}_{{\rm{i}}}\Delta {Q}_{{\rm{i}}}|}{{Q}_{\infty }}$$

where *t*_*i*_ is an intermediate time of the intervals of sampling time, *∆Q*_*i*_ is the amount of active pharmaceutical ingredient dissolved in every interval of *t* and *Q*_*∞*_ is the maximum of active pharmaceutical ingredient dissolved.

And t_50%_ (t_80%_) was the time necessary to release 50% (80%) of a drug tablet^43^.

To compare the release profiles, we calculated the similarity factor (*f*_2_) according to the formula of the FDA guideline “Dissolution Testing of Immediate Release Solid Oral Dosage Forms”. The similarity factor (*f*_2_) between two tablets, which is a logarithmic transformation of the sum-squared error of the differences between the dissolution curves, was obtained by^44^:

4 $${f}_{2}=50\times \,\log \{{[1+(\frac{1}{n})\mathop{\sum }\limits_{j=1}^{n}{|{R}_{j}-{T}_{j}|}^{2}]}^{-0.5}\times 100\}$$

where *n* is the number of time points in the dissolution profile, and *R*_*j*_ and *T*_*j*_ are the dissolution percentages at a specific time *j* of the two independent assays. In line with the guidelines, two dissolution profiles were regarded as similar when the *f*_2_ value was greater than 50.

## Results and Discussion

### Analytical method validation

The validation of analytical methodology is carried out according to FDA^45^ (Food and Drug Administration) guidelines, including accuracy, precision (repeatability and reproducibility), limit of detection (LOD), limit of quantitation (LOQ) and linearity. In these measurements, standard paracetamol or a mixture of standard chlorzoxazone and paracetamol is contained in the flow-through-cell, which is continuously delivered into the flow-gating interface followed by sequential injection and HSCE analysis. The optimal experimental conditions for validation are: sequential injection (gap distance: 50 μm; loading time: 2 s; injection time: 1 s; washout time: 2 s; sampling flow rate: 100 nL/min; BGE gating flow rate: 100 μL/min) and HSCE separation (separation voltage: −15 kV and BGE: 5 mM phosphate buffer (pH = 9) for one active ingredient component tablet; separation voltage: −12 kV and BGE: 10 mM phosphate buffer (pH = 9) for two active ingredient components tablet).

**Figure 2:**
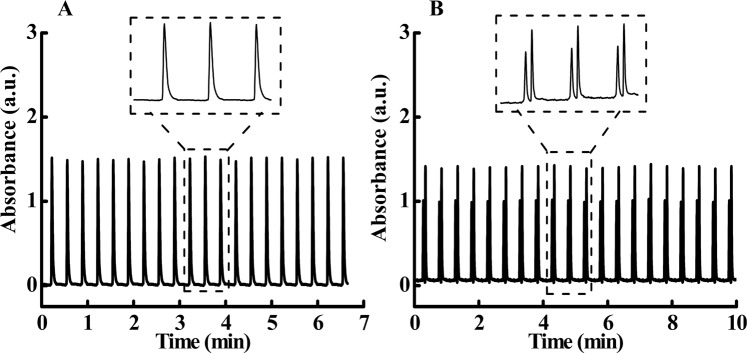
The repeatability of continuous sampling a 30-μg/mL standard solution of paracetamol (**A**) and a mixture of standard chlorzoxazone (50 μg/mL) and paracetamol (60 μg/mL) (**B**) using flow-through-cell coupling with flow gate injection under the optimum conditions (gap distance 50 μm, loading time 2 s, injection time 1 s, washout time 2 s, sampling flow rate 100 nL/min, BGE gating flow rate 100 μL/min).

Table S1 in the Supplementary Material shows the results of linear range, LOD and LOQ of the proposed method. It can be seen that the method presents a wide linear detection range with ppm-level LOD and LOQ for the detection of drug active ingredients. Figure 2 shows the electropherogram for twenty sequential analysis of each sample. For either of the samples, extremely reproducible peaks can be seen in each sequence (relative standard deviation, RSD (n = 20) < 0.93% for peak area). Moreover, efficient separation is achieved for each sequential sampling of the mixture sample, with RSD for the separation resolution less than 1.37% (see Fig. 2B).

The accuracy and precision of the system are evaluated by analyzing samples dosed by different amounts of paracetamol tablets. Twenty tablets were accurately weighed and ground into powder in a mortar. The amount taken from the powder, which was equal to 30% (0.15 mg), 80% (0.40 mg) or 120% (0.60 mg) of the weight of one tablet (0.5 mg), was dissolved in 100 mL of distilled water at 37.0 ± 0.5 °C for analysis by the system. We perform the experiments in three consecutive days. Table S2 in the Supplementary Material presents the results for inter-day and intra-day analysis. The recoveries of paracetamol and chlorzoxazone spiked with three different amounts of drug are determined to be in the range from 97.4% to 102.3% for each selected dose percentage, indicating good accuracy and precision of the proposed method.

### *In vitro* dissolution testing of drug tablets

**Figure 3:**
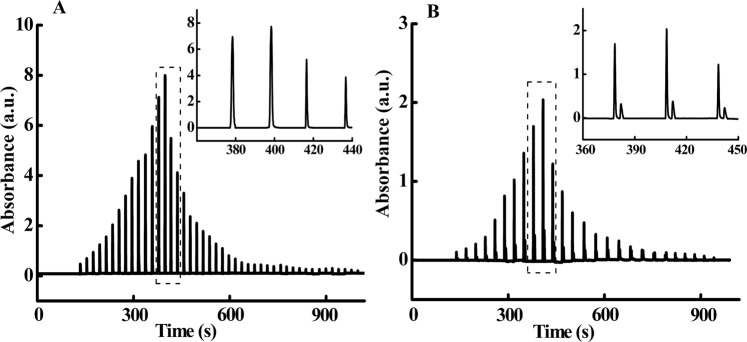
Electropherogram of a paracetamol immediate-release tablet (**A**) and a fixed-combination tablet containing chlorzoxazone and paracetamol (**B**) dissolved in ultrapure water using the Fig. 1 setup under optimal experimental conditions.

Immediate-release tablet containing either one or two active ingredients, i.e., a paracetamol tablet and a fixed-combination tablet containing chlorzoxazone and paracetamol, were used as examples to evaluate the feasibility of the proposed system for *in vitro* dissolution testing. In Fig. 3A,B, we present respectively typical electropherograms for the paracetamol immediate-release tablet and the fixed-combination chlorzoxazone tablet. Each flow gate injection is related to one time point of the dissolution process. A computer-controlled time program is used to control the temporal resolution of dissolution testing. As shown in those figures, the temporal resolution is 20 s for the paracetamol immediate-release tablet and that for the fixed-combination tablet is slightly higher (30 s) because HSCE separation is required in this case. As time progresses, the absorption peak gradually increases to a maximum and then begins to decrease until it eventually disappears after 15 minutes, indicating that the dissolution of the tablet in the flow-through-cell is complete. Hence, our method allows us to monitor the entire process of sequential dissolution with high-temporal resolution. Moreover, for the fixed-combination tablet, baseline separation of chlorzoxazone and paracetamol can be achieved in each sequence, as shown in the inset in Fig. 3B, which enables simultaneous and quantitative determination of the dissolution of both active pharmaceutical ingredients. The concentration of the analyte at each time point is obtained by the measured peak area and the calibration curve. The evolution of the concentration *vs* the dissolution time, i.e., the dissolution kinetic profile of the drug, can be achieved, as we discuss in the following section. Notably, the real dissolution time is calibrated from the retention time in the electropherogram by subtracting the time required for paracetamol (or chlorzoxazone) to migrate from the outlet of the flow-through-cell to the UV detection window of the capillary.

In the following section, we use the time-dependent dissolution percentage (*Dissolved %*), which is calculated by Eq. (1), to discuss and analyze the dissolution kinetics. The effect of the flow gate of the dissolution medium is investigated using a paracetamol immediate-release tablet for the test and distilled water as the dissolution medium and the results are presented in Supplementary Fig. S1 in the Supplementary Material. A faster dissolution rate is obtained at a greater flow rate. The percentage of dissolved drug at 10 min reaches 95% using a flow rate of 16 mL/min, while the percentages are 80% and 60% at flow rates of 8 mL/min and 4 mL/min, respectively. The flow rate of the dissolution medium is maintained at 16 mL/min in the following experiments.

#### Dissolution kinetic profiles in different media

**Figure 4:**
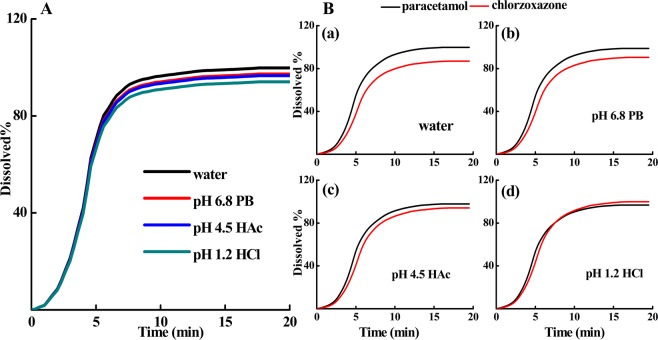
Comparison of the dissolution profiles of paracetamol immediate-release tablets (**A**) and fixed-combination chlorzoxazone tablets (**B**) in different dissolution media (water, pH 6.8 PB, pH 4.5 HAc, pH 1.2 HCl). Other conditions were the same as those in Fig. 3.

Dissolution testing is investigated in different media, which can influence drug solubility and the dissolution rate. According to the recommendation from *Ph. Eur*.^46^, four kinds of dissolution media that are commonly used as *in vitro* dissolution media were selected: (a) hydrochloric acid (HCl) with pH 1.2; (b) a pH 4.5 buffer solution consisting of acetic acid and sodium acetate; (c) a pH 6.8 buffer solution consisting of phosphate; and (d) distilled water. The dissolution kinetic curve of each medium is presented in Fig. 4A for the paracetamol immediate-release tablet and in Fig. 4B for the fixed-combination tablet. In Table 1, we show the resulting t_50%_, t_80%_, DE% and MDT values for both tablets. The results show that the dissolution is completed after 15 minutes, indicating the high dissolution rate of both tablets in any of the media. For paracetamol, the difference in the dissolution profile is not significant for different media, but the other active pharmaceutical ingredient, chlorzoxazone, dissolves much faster in a lower pH medium, which is clear from the simultaneous analysis of the components in the fixed-combination tablet, as shown in Fig. 4B.

**Table 1:** Drug release kinetics parameters for paracetamol immediate-release tablets (A) and fixed-combination chlorzoxazone tablets (B) using four types of dissolution media.

Media	Tablets	Active ingredients	t_50%_ (min)	t_80%_ (min)	DE%	MDT (min)
HCl (pH 1.2)	A	paracetamol	4.34	6.15	77.37	4.66
	B	paracetamol	5.35	7.96	68.60	5.83
		chlorzoxazone	5.80	7.99	61.15	7.75
Acetate (pH4.5)	A	paracetamol	4.30	5.89	75.44	4.59
	B	paracetamol	5.33	7.83	61.96	6.32
		chlorzoxazone	5.93	8.75	57.76	7.78
Phosphate (pH 6.8)	A	paracetamol	4.28	5.82	74.90	4.57
	B	paracetamol	5.30	7.72	62.58	7.26
		chlorzoxazone	6.04	9.50	55.35	7.80
Distilled water	A	paracetamol	4.25	5.57	72.96	4.49
	B	paracetamol	5.28	7.58	63.21	7.32
		chlorzoxazone	6.15	9.97	53.24	7.84

#### Similarity of the dissolution profiles

**Figure 5:**
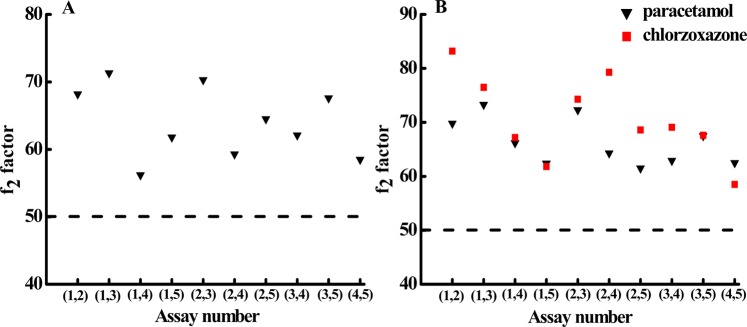
Scatter diagram of the *f*_2_ factor on paracetamol immediate-release tablets (**A**) and fixed-combination chlorzoxazone tablets (**B**) using the proposed method. In each figure, five independent assays are performed, and the *f*_2_ factor is presented between two assays as indicated in the parentheses on the *x*-axis. The total number (n) of data points in the dissolution profile for the calculation of each *f*_2_ are 45 for paracetamol immediate-release tablets and 30 for fixed-combination chlorzoxazone tablets. For fixed-combination tablets, the *f*_2_ factor is obtained for the dissolution profiles of both drug active ingredients, paracetamol (black triangle) and chlorzoxazone (red square). Other conditions are the same as those in Fig. 3.

The similarity of the dissolution profiles, which is evaluated by the *f*_2_ factor calculated by Eq. (4), is an important criterion for controlling and evaluating drug quality. High *f*_2_ values are associated with small average differences, and statistical similarity between the two curves requires 50 < *f*_2_ < 100. The scatter diagrams of the *f*_2_ values obtained using the proposed method for the immediate-release drug and the fixed-combination drug are presented in Fig. 5A,B, respectively. The *f*_2_ values are all greater than 50, indicating good similarity between any two dissolution profiles for the tablets of the immediate-release drug or the fixed-combination drug. The results also imply that the proposed method is reliable for accurate and repeatable drug dissolution testing.

#### Comparison to off-line assays

**Figure 6:**
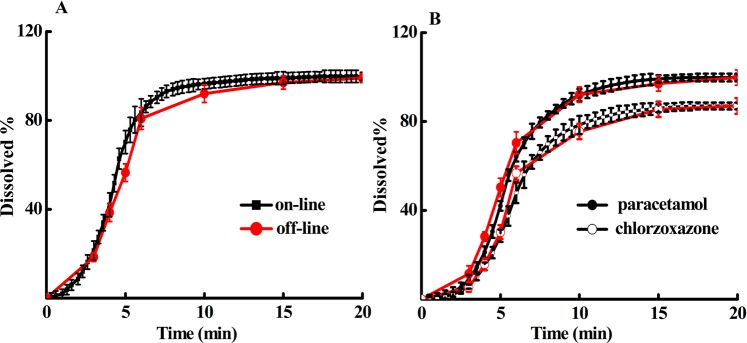
Comparison of the dissolution profiles using the proposed on-line automatic testing system and the traditional off-line testing method for paracetamol immediate-release tablets (**A**) and fixed-combination chlorzoxazone tablets (**B**). Other conditions were the same as those in Fig. 3.

We perform traditional off-line dissolution testing comparison with the results obtained using the proposed automatic testing system. The dissolution profiles are presented in Fig. 6A,B for immediate-release tablets and fixed-combination chlorzoxazone tablets, respectively. The overall dissolution profiles obtained by the off-line analysis and the automatic on-line analysis are in reasonable agreement with each other. The *f*_2_ factors are calculated to be 60.5 for paracetamol in the immediate-release tablets, and 63.4 and 58.9 for paracetamol and chlorzoxazone, respectively, in the fixed-combination tablets. The results indicate relatively good similarity of the dissolution profiles measured by the proposed method and the off-line analysis.

## Discussion

The proposed automatic on-line system that can measure the dissolution processes of drugs with high-temporal resolution. In the system, the sequential flow gate injection and on-line HSCE analysis are directly integrated with a flow-through-cell for drug dissolution; that is, eluted dissolution media containing dissolved active pharmaceutical ingredients from the flow-through-cell are sequentially injected at the flow-gating interface followed by efficient separation and sensitive detection via HSCE. The design of the entire system is very simple and easy-to-operate (see Fig. 1).

As demonstrated in the study, our method allows the entire process of drug release to be automatically monitored with high-temporal resolution (see Fig. 3) without any tedious and time-consuming procedures related to sample collection and analysis. These features are very important for obtaining accurate dissolution profiles, especially for immediate-release drugs. Dissolution testing is a time-dependent measurement of a kinetic process, thus high-temporal resolution should be required to monitor the process. As shown in Fig. 6A, the dissolution of the immediate-release tablet, paracetamol, is almost complete after six minutes. Clearly, if there was only four data points within the initial six minutes, as the results of the low-temporal resolved off-line measurements shown in Fig. 6A, it would be not accurate enough to understand the dissolution process, thus to control and evaluate drug quality, as well as to assess the quality and efficacy consistency of generic drugs.

In view of the statistical analysis of dissolution profile, the results would be more convincing with a larger number of sampling data (*n*). In our study, the similarity study between the on-line and off-line measurements (Fig. 6) is used to show the feasibility of the proposed method for dissolution testing. However, it should be mentioning that limited by the low-temporal resolution of the off-line measurements, quantitative evaluation of the two dissolution profiles is hard to determine. One can see that quite smooth curve can be obtained using the proposed on-line method with high-temporal resolution (see for guidance, the solid lines in Fig. 6).

The dissolution curve is essential to establish the *in vivo-in vitro* correlation (IVIVC), since the cost and risk of doing BE (bioequivalence) experiment is relatively high and the *in vitro* dissolution curve can show the intrinsic quality of the drug to some extent. Extracting the *in vivo* input profile, which directly corresponds to the *in vitro* dissolution profile, is one of the critical steps during establishing IVIVC. Monitoring the whole *in vitro* dissolution process in real time using, for example the proposed method with high-temporal resolution, is thus of great importance to reflect the *in vivo* performance of drugs.

The high-temporal resolution of the proposed method can be attributed to the program-controlled fast flow gate injection, which exhibits excellent reproducibility of the sequential analysis [see Fig. 2]; thus, the accuracy of drug dissolution testing is ensured. Moreover, with the advantages of HSCE for efficient separation and combined with high-speed analysis, the proposed method can simultaneously determine the dissolution processes of two or even more active pharmaceutical ingredients in fixed-combination drugs. The proposed method is not only used for dissolution testing of the ionized drug, but also for non-ionized drugs. For the analysis of non-ionized drugs, e.g. the active ingredients are neutral, the modified mode of CE, such as micellar electrokinetic capillary chromatography can be employed. The proposed approach will be of great value in analyses of the dissolution processes of fixed-combination drugs, especially those containing active ingredients with overlapping spectra, which are difficult to analyze by optical fiber or image-based *in situ* dissolution testing methods.

## Conclusions

In conclusion, we show that we can monitor dissolution profiles from the beginning to the end of the dissolution process for both immediate-release and fixed-combination drugs using the proposed method. Given the essential merits discussed above, including rapid and accurate analysis, high-temporal resolution, and the ability to perform fixed-combination drugs analysis, the proposed method is a promising approach that can be applied in a variety of pharmaceutical industry studies.

## Materials and Methods

### Reagents and materials

Immediate-release tablets (paracetamol, 500 mg/tablet, excipients including hydroxy propyl cellulose, pregelatinized starch, microcrystalline cellulose, povidone K-30, lauryl sodium sulfate, cassava starch, SiliciiDoxydum, magnesium stearate, sodium carboxymethyl starch, thiourea, NanJixue®, Guangdong, China) and fixed-combination tablets (chlorzoxazone, containing 125 mg chlorzoxazone and 150 mg paracetamol per tablet, excipients including sodium carboxymethyl starch, 3% hydroxypropyl methyl cellulose in aqueous solution, magnesium stearate, LunanBeite®, Shandong, China) were purchased from a local pharmacy and were used as examples to evaluate the performance of the proposed method. Sodium dihydrogen phosphate, disodium hydrogen phosphate, ammonium acetate, hydrochloric acid (HCl) and acetic acid (HAc) were obtained from Beijing Chemical Works (Beijing, China). All reagents and chemicals were analytical grade unless stated otherwise and were prepared in deionized water (18 MΩ·cm) using a Milli-Q Water System (Millipore, Bedford, MA, USA) and filtered with a 0.22-µm membrane filter prior to use.

Fused silica capillaries (Hebei Yongnian Optical Fiber Factory, Hebei, China) with an inner diameter (i.d.) of 50 µm and an outer diameter (o.d.) of 365 µm were used. The separation capillary was pretreated according to standard methods for 10 min with 0.1 M NaOH, 10 min with deionized water and then with the BGE. The BGE was a 5 mM phosphate buffer (pH 9.0).

### Developing the automatic dissolution testing system

In order to fulfill the pharmacopoeial requirements, the flow-through dissolution apparatus was designed and manufactured according to the pharmacopoeial requirements of UPS for flow-through dissolution apparatus (USP apparatus IV). A vertically positioned flow-through-cell, which was home-made as a vertical cylinder (a diameter of 22.6 mm or a cross-sectional area of 4 cm^2^) with a 40°conical portion (a volume of approximately 4.2 cm^3^), was placed in a water bath to control the temperature at 37 ± 0.5 °C. The conical portion of the cell was connected to a reservoir tank containing the dissolution medium, which was also maintained at 37 ± 0.5 °C. A piston pump (HL-300, Shanghai JingKe Industry Co., Ltd., Shanghai, China) was used to force the dissolution medium upward through the vertically positioned flow-through-cell with a typical flow rate of 4, 8 or 16 mL/min. A tablet was positioned vertically half-way up the cell supported by a stainless-steel holder. Glass beads (1-mm diameter, a total weight of 6.4 g) filled the conical portion of the cell, which could produce laminar flow conditions as the release medium was driven into the cell to release the tablet. A larger glass bead with a 5-mm diameter was placed at the cone apex to prevent any material from descending into the inlet tubing. The dissolution medium that carried the dissolved active pharmaceutical ingredients of the drug continuously flowed out of the upper end of the cell through two filter membranes (Glass Microfibre Filters 25 mm, Whatman^TM^) with respective pore diameters of 2.7 µm (GF/D filter) and 0.7 µm (GF/F filter). The eluent was divided by a sample splitter through back pressure produced by a computer-controlled pressure valve (FV420320, Yueqing Huachi Pneumatic Components co., Ltd), allowing it to enter the sampling capillary (5-cm length) with a flow rate of 100 nL/min.

Automatic sequential injection of the eluted sample was performed with a flow-gating interface, which was produced in-house from a commercial four-channel Microcross Assembly (Upchurch Scientific, Oak Harbor, WA). The end of the sampling capillary and the injection end of the separation capillary (20-cm total length) were tapered, sleeved with micro-tight sleeves (0.015-inch i.d. and 0.025 -inch o.d.), and then tightly inserted into two opposite channels of the interface with removable nuts and ferrules. The distance between the ends of the capillaries (i.e., the injection space) in the interface was approximately 50 µm (see the inset images in Fig. 1A). The remaining two channels of the interface, which were drilled to expand the diameters, were fitted with PTFE tubes (4-mm in o.d., 0.8-mm i.d.) for delivering and draining gating buffer solution from the injection space. Continuous BGE (5 mM phosphate buffer, pH 9.0) flow was kept constant at 100 µL/min for all measurements, which was controlled by a syringe pump (Longer Precision Pump Co., Ltd, Hebei, China). The flow-gating interface was grounded, and negative high-voltage was applied at the outlet of the separation capillary for HSCE. By using high electric field strengths, short separation distances, and narrow sample plugs, HSCE exhibits a rapid separation speed and high separation efficiency compared to traditional CE techniques. For HSCE separation and detection, the effective length of the separation capillary was kept as short as 5 cm, and the separation electric potential was 750 V/cm. The detection wavelength was 257 nm for paracetamol immediate-release tablets and 220 nm for fixed-combination chlorzoxazone tablets.

To optimize the experimental conditions for automatic sequential sample injection with the flow-gating interface, the outlet end of the sampling capillary was directly connected to a syringe pump. A standard sample containing the active pharmaceutical ingredients with a desired concentration was continuously delivered into the flow-gating interface through the sampling capillary.

### Evaluation of sequential injection and HSCE separation

Supplementary Fig. S2 shows electropherograms of HSCE under various conditions in which a 30 μg/mL standard paracetamol sample is used for analysis.

In the flow-gating interface, utilizing the cone-shaped ends of the tapered capillaries benefits the performance of the interface. Two tapered capillaries (the sampling capillary and separation capillary) were precisely positioned and aligned under a microscope to facilitate efficient sample transfer. The gating extent of the sample is determined by the gap distance and the rates of the sampling flow and BGE gating flow. In Supplementary Fig. S2-a, we show electropherograms recorded under three different gap distances between the tapered capillaries in the flow-gating interface. Clearly, with a small gap distance of 10 µm, double peaks appear for HSCE of paracetamol due to sample leaking from the sampling capillary into the separation capillary during CE. On the other hand, if the gap distance is too large (100 µm in the present study), dilution of the sample in the buffer in the injection space is so severe that very little sample will be transferred into the separation capillary for CE, resulting in no signal in the electropherogram, as shown in Supplementary Fig. S2-a. With the gap distance at 50 µm, we investigate the effects of the BGE gating flow rate and the sampling flow rate, and the results are shown in Supplementary Fig. S2-b,c. In principle, a higher BGE flow rate would more effectively prevent the sample from leaking into the separation capillary during HSCE. However, a bottleneck effect will occur if the BGE flow is too high, leading to concurrent washing out of the analyte in the separation capillary during the washing time, thus reducing the sampling efficiency. As shown in Supplementary Fig. S2-b, relatively constant peak heights are obtained at BGE flow rates between 20 and 100 μL/min. Considering that a low gate flow will result a longer time to wash out the external sample, a BGE gate flow rate of 100 μL/min was selected. Electropherograms of flow gate injection at different sampling flow rates are shown in Supplementary Fig. S2-c. A low sampling flow (<100 nL/min) will decrease the injection amount of sample for a specific loading time (e.g., 2 s in the analysis). In the present analysis, constant peak areas were observed when the sampling flow rates exceeded 100 nL/min, indicating a required minimal appropriate sampling flow rate.

The running sequence (loading, washing and injection times) is critical for the proposed method. In the flow-gating interface, the injection space is small and may require a short washing time to “wash out” the sample by the BGE. As shown in Supplementary Fig. S2-d, a similar peak width and symmetry can be obtained with a washout time of 2 s or 3 s. A washing time that is too short, however, may not allow efficient washing out of the sample in the injection space, leading to the appearance of double peaks (see the result of 1-s washing time in Supplementary Fig. S2-d). The loading and injection times determine accumulation and injection, as well as longitudinal diffusion of the sample plug, and will thus affect the peak height and symmetry of HSCE. With a washing time of 2 s, the loading and injection times are investigated and optimized to be 2 s and 1 s, respectively, as shown in Supplementary Fig. S2-e,f.

To optimize the parameters involved in HSCE, including the buffer concentration, the buffer pH and the separation voltage, separation of a mixture of standard chlorzoxazone (50 μg/mL) and paracetamol (60 μg/mL) was investigated. As shown in Supplementary Fig. S3, by increasing the buffer concentration in the range of 5–25 mM, the migration time increases and the separation resolution of the analytes is slightly enhanced. Additionally, increasing the pH value of the buffer from 6 to 9 results in a decreased migration time and a slightly reduced separation resolution. For the separation voltage, a higher voltage leads to more rapid analysis but a lower separation resolution. Using a 10 mM BGE buffer at pH 9 and 12-kV separation voltage, baseline separation of chlorzoxazone and paracetamol can be achieved in less than 30 s with the theoretical plates of 2.8 × 10^5^ and a separation resolution of 1.8. Considering the dissolution testing results for immediate-release paracetamol tablets, a 30-μg/mL standard solution of paracetamol was used to optimize the above experimental conditions for HSCE detection. The results are shown in Supplementary Fig. S4. In less than 20 s, nearly 4.3 × 10^5^ theoretical plates were achieved under the optimized HSCE conditions (5 mM BGE buffer at pH 9 and 15-kV separation voltage).

### Traditional off-line dissolution testing

For traditional off-line dissolution testing of immediate-release drug or the fixed-combination drug, the eluted medium carrying the dissolved drug from the flow-through-cell was collected as the entire outflow over the sampling point of 3, 4, 5, 6, 10, 15, 20 min. The concentration of the drug in each fraction was determined using a CE-UV system at a separation electric potential of 400 V/cm, with 5 mM phosphate buffer (pH 9.0) as a running buffer. The sample was injected at a height of 10 cm for 5 s. The detection wavelength was 257 nm for paracetamol immediate-release tablets and 220 nm for fixed-combination chlorzoxazone tablets. For quantitative analysis, the calibration curves of the drugs were drawn by plotting the peak area versus the concentration in a range of 0–600 μg/mL for paracetamol and 0–500 μg/mL for chlorzoxazone.

## Supplementary information

Automatic Dissolution Testing with High-Temporal Resolution for Both Immediate-Release and Fixed-Combination Drug Tablets

## Data Availability

The datasets generated during and/or analysed during the current study are available from the corresponding author on reasonable request. All data generated or analysed during this study are included in this published article (and its Supplementary Information Files).
