# Supplementary material for: Automatic Dissolution Testing with High-Temporal Resolution for Both Immediate-Release and Fixed-Combination Drug Tablets
Source: Sci Rep. 2019 Nov 19;9:17114. doi: 10.1038/s41598-019-53750-w (PMC6863837; doi:10.1038/s41598-019-53750-w)
Supplement: Supplementary file 1 — Automatic Dissolution Testing with High-Temporal Resolution for Both Immediate-Release and Fixed-Combination Drug Tablets [file 41598_2019_53750_MOESM1_ESM.pdf]

Supplementary Information for

Automatic Dissolution Testing with High-Temporal Resolution for Both Immediate-Release and Fixed-Combination Drug Tablets

Zhongmei Chi[1], Irfan Azhar[1], Habib Khan[1], Li Yang*[1]

[1]Faculty of Chemistry, Northeast Normal University, 5268 Renmin Street, Changchun, Jilin,130024, P. R. China

Yunxiang Feng[2]

[2]Jingke-Oude Science and Education Instruments Co., Ltd., Changchun, Jilin, 130024, P. R. China

* Corresponding author:

L. Yang, yangl330@nenu.edu.cn, Tel: +86-431-85099762, Fax: +86-431-85099762

Results and discussion

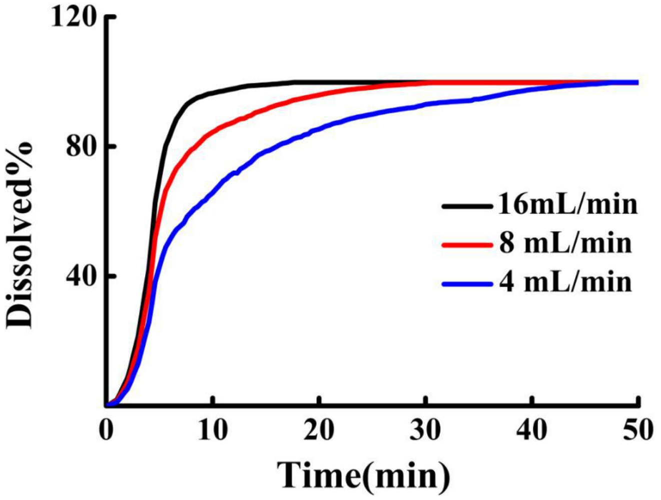

Figure S1. Effect of the flow-through-cell flow rate on paracetamol immediate-release tablet dissolution profiles is obtained by flow-through-cell coupling with the flow gate injection method in ultrapure water at a flow rate of 4 mL/min, 8 mL/min, and 16 mL/min. Each value represents the mean ± SD (n = 3).

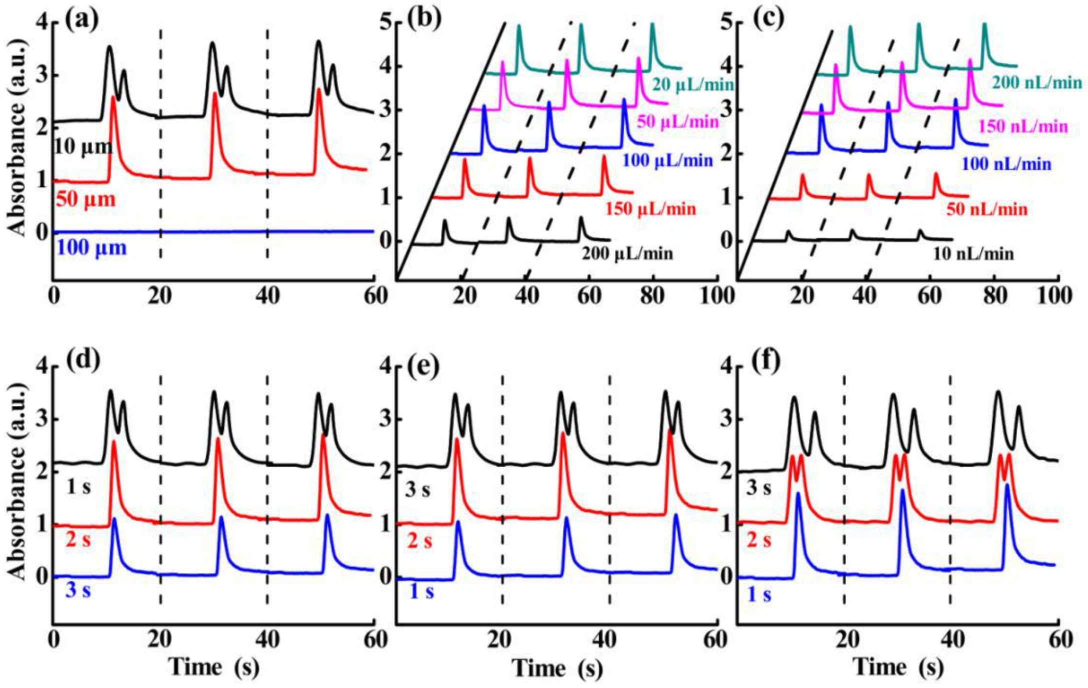

Figure S2. Electropherograms of HSCE under various conditions. (a) Gap distance; (b) BGE gating flow rate; (c) sampling flow rate; (d) washing time; (e) loading time; (f) injection time. A 30-µg/mL standard paracetamol sample prepared in BGE was used for analysis.

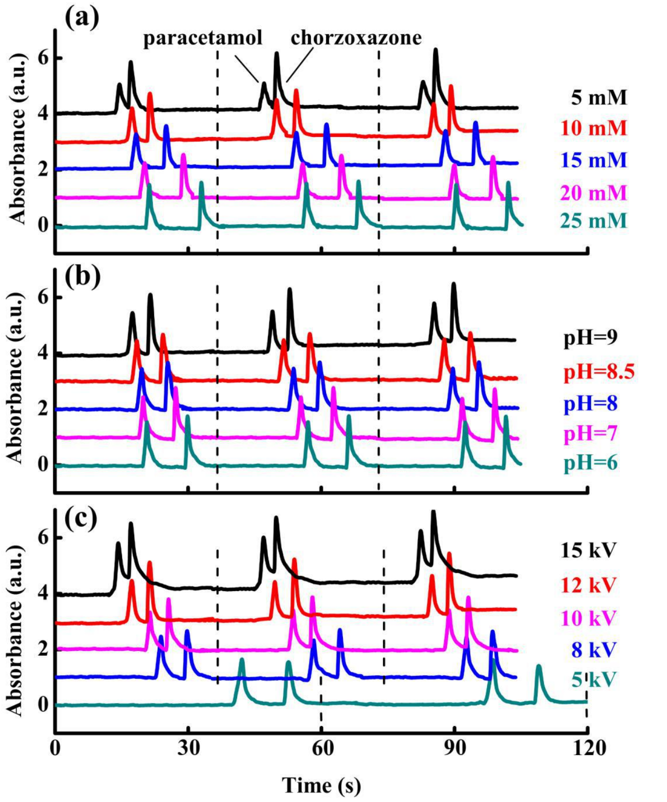

Figure S3. Electropherograms of standard chlorzoxazone and paracetamol under different HSCE conditions. (a) BGE buffer concentration; (b) BGE buffer pH; (c) separation voltage. A mixture of standard chlorzoxazone (50 μg/mL) and paracetamol (60 μg/mL) prepared in BGE was used for analysis. Other experimental conditions are the same as those in Figure S2.

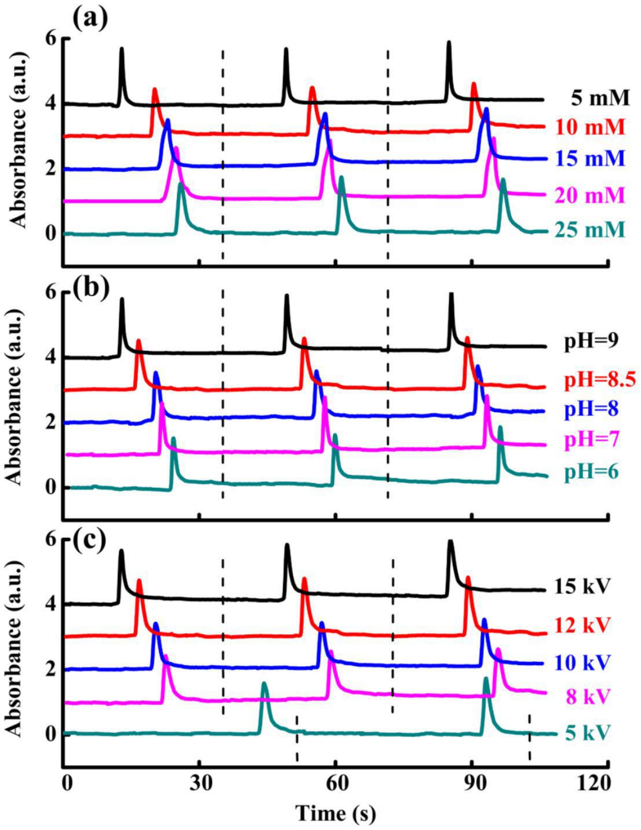

Figure S4. Electropherograms of standard paracetamol under various HSCE conditions (a) BGE buffer concentration; (b) BGE buffer pH; (c) separation voltage. Standard paracetamol (30 µg/mL) prepared in BGE was used for analysis. Other experimental conditions are the same as those in Figure S2.

Table S1. Parameters of the linear dependences of the peak areas on the concentrations of analytes.

Analytical Parameter	Paracetamol	Mixture solution	
		Paracetamol	Chlorzoxazone
Linear dependences	Y=0.4160X+0.6689	Y=0.2492X+1.5287	Y=0.4696X+0.6812
R^2	0.9991	0.9989	0.9990
Linear range (µg/mL)	1-600	2-600	1-500
LOD (µg/mL)	0.3	0.6	0.4
LOQ (µg/mL)	1.0	2.0	1.0

The limit of detection (LOD) and the limit of quantitation (LOQ) determined by the assays (LOD = 3 *s/m*, LOQ = 10 *s/m*, where *s* is the standard deviation of the blank (n = 5) and *m* is the slope of the corresponding calibration curve).

Table S2. Accuracy and precision data for the determination of paracetamol tablets.

Added (mg)	Repeatability (within-day, n = 3)			Reproducibility (between-day, n = 5)		
	Found (mg)	RSD (%)	Recovery (%)	Found (mg)	RSD (%)	Recovery (%)
0.15	0.1517	1.25	101.1	0.1483	1.96	98.9
0.40	0.3897	2.16	97.4	0.3926	1.01	98.2
0.60	0.6026	1.77	100.4	0.6122	2.14	102.3

RSD: Relative standard deviation.
